# Antibiotic Resistance Profiles and Molecular Mechanisms of *Campylobacter* From Chicken and Pig in China

**DOI:** 10.3389/fmicb.2020.592496

**Published:** 2020-10-27

**Authors:** Mengjun Tang, Qian Zhou, Xiaoyan Zhang, Sheng Zhou, Jing Zhang, Xiujun Tang, Junxian Lu, Yushi Gao

**Affiliations:** Jiangsu Institute of Poultry Sciences, Supervision, Inspection and Testing Centre for Poultry Quality (Yangzhou), Ministry of Agriculture, Yangzhou, China

**Keywords:** *Campylobacter*, antimicrobial resistance, resistance mechanism, chicken, pig

## Abstract

The purpose of this research was to characterize the antibiotic resistance profiles of *Campylobacter* spp. derived from chicken and pig feces collected from farms in Jiangsu Province, China, and to analyze the relevant resistance mechanisms among antimicrobial-resistant *Campylobacter* spp. isolates. Antibiotic susceptibility to nine antibiotic agents was tested with the microdilution method in 93 *Campylobacter* spp. (45 *C. jejuni* and 25 *C. coli* from chickens; 23 *C. coli* from pigs). High rates of resistance were observed to nalidixic acid (79.6%), erythromycin (75.3%), tetracycline (68.8%), azithromycin (66.7%), ciprofloxacin (64.5%), and gentamicin (35.5%), with a lower resistance rate to florfenicol (8.6%). The prevalence of the tested antibiotic resistance in *C. coli* was higher than in *C. jejuni* from chickens. The rate of antimicrobial resistance to ciprofloxacin in *C. coli* isolates from chickens was 100.0%, and the *C. coli* isolates from pigs were all resistant to erythromycin (100%). Most of *C. jejuni* (64.4%) and *C. coli* (64.5%) isolates displayed multi-drug resistance. All the *Campylobacter* spp. isolates resistant to fluoroquinolones had the C257T mutation in the *gyr*A gene. All 64 tetracycline-resistant *Campylobacter* spp. isolates were positive for the *tet*O gene. The *tet*A gene was also amplified in 6.5% of *Campylobacter* spp. isolates, whereas *tet*B was not detected among the isolates. The A2075G point mutation in the 23S rRNA gene occurred in 86.1% (62/72) of the macrolides-resistant *Campylobacter* spp. isolates, and the *erm*B gene was identified in 49 *Campylobacter* spp. isolates (30 *C. jejuni* and 19 *C. coli*). Amino acid insertions or mutations in the L4 and L22 ribosomal proteins were not linked to macrolide resistance. These results highlight the high prevalence of resistance to multiple antibiotics, particular macrolides, among *Campylobacter* spp. from chickens and pigs in Jiangsu Province, China, which is probably attributable to the overuse of antimicrobials in chicken and pig production. These findings recommend the more cautious use of critical antimicrobial agents in swine and poultry production. Stringent and continuous surveillance is required to reduce the drug-resistant campylobacteriosis in food animals and humans.

## Introduction

Campylobacteriosis is an essentially foodborne zoonotic disease worldwide (Allos, 2001). Most of these human illnesses involve *C. jejuni* (81%) or *C. coli* (8.4%) (Sifré et al., 2015). Enteric diseases caused by the thermophilic species *C. jejuni*, *C. coli*, *C. lari*, and *C. upsaliensis* range from asymptomatic infections to severe inflammatory bloody diarrhea (Ketley, 1997). In addition, *C. jejuni* is often associated with the Guillain-Barre syndrome (Dingle et al., 2001). *Campylobacter* spp. widely and asymptomatically colonize the intestinal tracts of food producing animals (Altekruse et al., 1999; Harvey et al., 1999; Stanley and Jones, 2003), and *C. jejuni* is the main species affecting chickens, whereas *C. coli* is mainly found in pigs (Sáenz et al., 2000; Haruna et al., 2013). Poultry with no obvious clinical symptoms are the main reservoirs of these pathogens (Luangtongkum et al., 2006; Humphrey et al., 2007). Pigs are also usually subclinically infected with *Campylobacter* spp. During the slaughter process, intestinal content spillage could contaminate the meat and meat products (Weijtens et al., 1997; Gill et al., 1999).

Although *Campylobacter* infection is usually self-limiting, severe, long-lasting or systemic *Campylobacter* infections can be treated with antibacterial drugs (Allos, 2001). Tetracyclines, macrolides, and fluoroquinolones are used to control *Campylobacter*-mediated infections (Sifré et al., 2015). In food-producing animal industries, ciprofloxacin (a fluoroquinolone) is frequently used because it has a broad antibacterial spectrum. In recent decades, fluoroquinolone resistance in animal and human *Campylobacter* isolates have increased worldwide (Engberg et al., 2001; Moore et al., 2006; Wang et al., 2016). With this increase in fluoroquinolone-resistant *Campylobacter*, macrolides have become common drugs in treatment of campylobacteriosis (Luangtongkum et al., 2009). Macrolides, including tylosin, tilmicosin, tulathromycin, and tildipirosin, have been widely used in animal production in many countries (Poehlsgaard et al., 2012; McEwen and Collignon, 2018), and macrolide-resistant *Campylobacter* strains have been isolated in many regions (Kaakoush et al., 2015; Zhang et al., 2016) and from animals (Wang et al., 2016; Lim et al., 2017; Hafez and Attia, 2020). Tetracycline has also been extremely widely used in animal farming because it has broad-spectrum antibacterial activity, low cost, and excellent efficacy (Chopra and Roberts, 2001), and it may have cause high tetracycline resistance in *Campylobacter* from food-producing animals (Premarathne et al., 2017; Woźniak-Biel et al., 2018). The proportion of *Campylobacter* spp. strains resistant to ciprofloxacin, tetracycline, and erythromycin is increasing (García-Fernández et al., 2018). A tendency to detect increasing multidrug-resistant (MDR) strains has been reported in both humans (Feizabadi et al., 2007) and food-producing animals (Li et al., 2016; Neogi et al., 2020). The potential risk of MDR *Campylobacter* spread from animals to human is of great concern.

In *Campylobacter*, the C257T mutation in the gyrase gene (*gyr*A) is the most frequent mechanism causing quinolone and fluoroquinolone resistance (Iovine, 2013). Efflux pumps and ribosomal protection genes are two major mechanisms of tetracycline resistance in various bacteria (Roberts, 2005). The *tet*O gene encodes a ribosomal protection protein that is primarily responsible for tetracycline-resistance. It is carried on the chromosome (Chopra and Roberts, 2001) or transferred on plasmids (Gibreel et al., 2004). The efflux genes, *tet*A and *tet*B, encode membrane-bound efflux proteins that export tetracycline from the cell (Chopra and Roberts, 2001), and the presence of *tet*A in *Campylobacter* isolates was first detected by Abdi-Hachesoo et al. (2014). *Campylobacter* has three main mechanisms that confer resistance to macrolides: modifications to ribosome-associated genes (V region of 23S rRNA and the *rpl*D and *rpl*V ribosomal-protein-encoding genes) (Luangtongkum et al., 2009), multidrug efflux pumps mediated by *Cme*ABC (Lin et al., 2002), and a ribosomal methylase encoded by *erm*B, which was first reported in China in 2014 (Qin et al., 2014). Since then, *erm*B-positive *Campylobacter* has been isolated from broilers in Spain and the United States (Florez-Cuadrado et al., 2016).

Several studies have been published in China that report relatively high levels of antimicrobial resistance in *Campylobacter* from animals in different areas (Ma et al., 2014, 2017). Furthermore, in a campylobacteriosis outbreak in China in 2018, and the domain isolate was resistant to nalidixic acid, tetracycline and ciprofloxacin (Qu et al., 2019). In China, a national annual antimicrobial-resistance surveillance system for *Campylobacter* in food-producing animals has been in place from 2017 to 2020 to strengthen the monitoring of antimicrobial resistance and ensure the safety of feed animals and public health. To obtain epidemiological data on *Campylobacter* from food-producing animals in Jiangsu Province, China, the resistance rates of *Campylobacter* strains in chicken and pig feces on farms and the relevant molecular mechanisms of their resistance to quinolones, tetracycline, and macrolides have been investigated.

## Materials and Methods

### Sample Collections, Bacterial Isolation, and Identification

A total of 150 chicken cloacal swabs were collected on 15 chicken farms (seven layer farms and eight broiler farms), and 100 pig feces samples were collected on 10 pig farms, all located in five cities of Jiangsu Province, China. These cities (Changzhou, Suqian, Nantong, Yancheng, and Yangzhou) are the major food animal production areas for chickens and pigs in Jiangsu Province. Ten cloacal swabs or fresh feces samples were taken from randomly selected animals on each farm. After the samples were collected, they were transported to the laboratory under refrigerated conditions within 24 h. The collected samples were diluted with phosphate-buffered saline (PBS). After dilution, 50 μL of the appropriate concentration diluent was streaked onto *campylobacter* blood-free selective agar base (modified CCDA-preston; Oxoid CM1183, United Kingdom) containing cefoperazone (LKT, C1630) and amphotericin B (Wako, 011-1363). The agar plates were incubated at 42°C for 36 h under a microaerophilic atmosphere containing 85% N_2,_ 10% CO_2_ and 5% O_2_. The suspected colonies were subcultured on Mueller-Hinton agar (MH, Difco MD) containing 5% defibrinated sheep blood under microaerophilic conditions at 42°C for 36 h, and confirmed with multiplex PCR, as described previously (Huang et al., 2009). The primers used are listed in Table 1. All the positive isolates were stored in brain-heart infusion (BHI) broth with 20% glycerol at −80°C.

**TABLE 1:** List of primers used for PCR in this study.

**Gene**	**Primer sequence (5’-3’)**	**Product size (bp)**	**References**
16s rRNA	F: ATCTAATGGCTTAACCATTAAAC R: GGACGGTAACTAGTTTAGTATT	857	Huang et al., 2009
*map*A	F: CTATTTTATTTTTGAGTGCTTGTG R: GCTTTATTTGCCATTTGTTTTATTA	589	Huang et al., 2009
*ceu*E	F:AATTGAAAAATTGCTCCAACTATG R:TGATTTTATTATTTGTAGCAGCG	462	Huang et al., 2009
23S rRNA	F: GTAAACGGCGGCCGTAACTA	699	Jensen and Aarestrup, 2001
	R: CATCCATTACACACCCAGCC		
*rpl*V	F: GAATTTGCTCCAACACGC	520	Cagliero et al., 2006
	R: ACCATCTTGATTCCCAGTTTC		
*rpl*D	F: GTAGTTAAAGGTGCAGTACCA	740	Cagliero et al., 2006
	R: GCGAAGTTTGAATAACTACG		
*erm*B	F:TGAAAAAGTACTCAACCAAAT	692	Qin et al., 2014
	R:TCCTCCCGTTAAATAATAGAT		
*gyr*A	F:ATTTTTAGCAAAGATTCTGAT	673	Zirnstein et al., 1999
	R: CCATAAATTATTCCACCTGT		
*tet*O	F:AACTTAGGCATTCTGGCTCAC	515	Abdi-Hachesoo et al., 2014
	R: TCCCACTGTTCCATATCGTCA		
*tet*A	F: GTGAAACCCAACATACCCC	888	Abdi-Hachesoo et al., 2014
	R: GAAGGCAAGCAGGATGTAG		
*tet*B	F: CCTTATCATGCCAGTCTTGC	774	Abdi-Hachesoo et al., 2014
	R: ACTGCCGTTTTTTCGCC		

### Antibiotic Susceptibility Testing

The minimum inhibitory concentrations (MICs) of antimicrobial agents for *Campylobacter* were assessed with the broth microdilution method, using commercially available Sensititre^®^ susceptibility plates for *Campylobacter* (TREK Diagnostic Systems, East Grinstead, United Kingdom), according to the manufacturer’s instructions. Nine antimicrobial agents belonging to seven classes were tested: fluoroquinolones (ciprofloxacin [CIP] and nalidixic acid [NAL]), macrolides (erythromycin [ERY] and azithromycin [AZM]), ketolides (telithromycin [TEL]), tetracyclines (tetracycline [TET]), aminoglycosides (gentamicin [GEN]), phenicols (florfenicol [FFN]), and lincosamides (clindamycin [CLI]). The MIC breakpoints for resistance were interpreted according to the NARMS-2014 recommendations for *Campylobacter*. *C. jejuni* strain ATCC 33560 and Mueller-Hinton broth with TES buffer and lysed horse blood were used as the positive control and the negative control, respectively. The results can be considered satisfactory if QC results are within range. To ensure the reproducibility of the MIC data, assays were repeated twice, each in duplicate. Multi-drug resistance was defined as resistance to three or more antimicrobial classes.

### Preparation of Genomic DNA

All genomic DNA of the *Campylobacter* isolates were extracted with the TIANamp Bacterial DNA Kit (Tiangen, Beijing, China), according to the manufacturer’s protocol, and stored at −20°C.

### Molecular Biological Methods for Detecting Antimicrobial Resistance

Ciprofloxacin resistance: The fluoroquinolone-resistance-determining region (QRDR) of the *gyr*A gene was amplified with PCR, as previously reported by Zirnstein et al. (1999). The PCR product of 673 bp was sequenced and compared with the sequences of the *gyr*A gene of *Campylobacter* obtained from GenBank.

Tetracycline resistance: Three tetracycline resistance genes (*tet*A, *tet*B, and *tet*O) were investigated in the *Campylobacter* isolates with PCR, as previously described (Abdi-Hachesoo et al., 2014).

Erythromycin resistance: region V of 23S rRNA (Jensen and Aarestrup, 2001), the ribosomal protein L4 gene *rpl*D, and the ribosomal protein L22 gene *rpl*V were amplified with PCR (Cagliero et al., 2006). The PCR products were then directly sequenced at Sangon Biotech (Shanghai, China). The DNA sequences obtained were compared with the sequence of the *C. jejuni* NCTC 11168 genome to identify specific mutations that had occurred in the ribosome. To test the ribosomal RNA methylase gene, *erm*B, it was amplified as reported by Qin et al. (2014). The oligonucleotide sequences of the primers and the sizes of the PCR products are given in Table 1.

### Statistical Analysis

The percentage differences in resistance to the nine tested antimicrobial agents between the *C. jejuni* and *C. coli* isolates were analyzed with the χ^2^ test in SAS 9.2 (SAS Institute Inc., Cary, NC, United States). *P* < 0.05 was considered statistically significant.

### Ethics Statement

This study was performed according to the guidelines of the Animal Welfare and Ethical Censor Committee at Jiangsu Institute of Poultry Science. No chickens or pigs were sacrificed for the present study. When collecting cloacal swabs, well-trained farm workers held the chickens. Fresh feces from pigs were collected without any manipulation of the pigs.

## Results

### Isolation and Identification of *Campylobacter* spp.

A total 93 *Campylobacter* strains were isolated and identified from the 250 samples collected, with a total isolation rate of 37.2%. The occurrence of *Campylobacter* in the food animals ranged from 0 to 57.5%. Among them, 45 *C. jejuni* and 25 *C. coli* were isolated from chickens. The occurrence of *Campylobacter* was higher in the broilers (57.5%) than in the layers (34.3%, *P* < 0.0001). Twenty-three of the *Campylobacter* isolates from pigs were identified as *C. coli* (Table 2).

**TABLE 2:** Prevalence of *Campylobacter* strains isolated from chicken and pig in Jiangsu Province, China.

**Sources**		No. of samples*	**No. of *Campylobacter* strains (isolation rate %)**		
			***C. jejuni***	***C. coli***	**Total**
Chicken	Broiler	80	32(40.0)	14(17.5)	46(57.5)
	Layer	70	13(18.6)	11(15.7)	24(34.3)
Pig		100	0(0)	23(23.0)	23(23.0)
Total		250	45(18.0)	48(19.2)	93(37.2)

*No. of samples were 10 per farm.

### Antimicrobial Resistance

The antimicrobial resistance to the nine tested antimicrobial agents in these *Campylobacter* isolates are given in Table 3. High rates of resistance were observed to nalidixic acid (79.6%), erythromycin (75.3%), tetracycline (68.8%), azithromycin (66.7%), ciprofloxacin (64.5%), and gentamicin (35.5%) in the *Campylobacter* spp. strains, with a lower resistance rate to florfenicol (8.6%). Overall, the prevalence of resistance to the antibiotics tested was higher in *C. coli* than in *C. jejuni* from chickens. Resistance to fluoroquinolones was highest (80%–100%) in *Campylobacter* from chickens, except for ciprofloxacin in *C. jejuni.* The second highest rate of resistance was to tetracyclines, followed resistance to macrolides. The resistance rates of the *C. jejuni* (66.7%) and *C. coli* isolates (100.0%) from chickens to ciprofloxacin differed significantly (*P* = 0.0011). However, there was no significant difference between the resistance rates of the *C. jejuni* and *C. coli* isolates from chicken to the other antimicrobial agents. Resistance to erythromycin (100%) and azithromycin (82.6%) was much higher in the *C. coli* isolates from pigs than in *C. coli* (76.0%, *P* = 0.012 and 64.0%, *P* = 0.1472, respectively) or in *C. jejuni* (62.2%, *P* = 0.0007 and 60.0%, *P* = 0.05942, respectively) from chickens. The rates of resistance to ciprofloxacin (21.7%), nalidixic acid (65.2%), and tetracycline (43.5%) in the *C. coli* isolates from pigs were lower than in the *C. coli* isolates from chickens (100.0%, *P* < 0.0001, and 92.0%, *P* = 0.0225, and 88.0%, *P* = 0.0011, respectively).

**TABLE 3:** The rate of antimicrobial resistance in *C. jejuni* and *C. coli* isolated from chicken and pig samples.

**Antimicrobial agents**	**Antibiotic breakpoints (μg/mL)**	**Total % (*n* = 93)**	**Source of isolate and number of resistant isolates**			
			**Chicken**		**Pig**	
			***C. jejuni* % (*n* = 45)**	***C. coli* % (*n* = 25)**	***C. jejuni* % (*n* = 0)**	***C. coli* % (*n* = 23)**
**Macrolides**						
Erythromycin	≥ 32	75.3(70)	62.2(28)	76.0(19)	0(0)	100.0(23)
Azithromycin	≥ 8	66.7(62)	60.0(27)	64.0(16)	0(0)	82.6(19)
**Ketolides**						
Telithromycin	≥ 16	47.3(44)	42.2(19)	44.0(11)	0(0)	60.9(14)
**Fluoroquinolones**						
Nalidixic acid	≥ 64	79.6(74)	80.0(36)	92.0(23)	0(0)	65.2(15)
Ciprofloxacin	≥ 4	64.5(60)	66.7(30)	100.0(25)	0(0)	21.7(5)
**Aminoglycosides**						
Gentamicin	≥ 8	35.5(33)	31.1(14)	40.0(10)	0(0)	39.1(9)
**Phenicols**						
Florfenicol	≥ 8	8.6(8)	6.7(3)	8.0(2)	0(0)	13.0(3)
**Tetracyclines**						
Tetracycline	≥ 16	68.8(64)	71.1(32)	88.0(22)	0(0)	43.5(10)
**Lincosamides**						
Clindamycin	8	56.9(53)	62.2(28)	64.0(16)	0(0)	39.1(9)

In this study, 88 out of the examined 93 isolates (94.6%) were resistant to at least one antimicrobial agent, whereas five *C. jejuni* isolates (5.4%) were susceptible to all the antimicrobial agents tested. Resistance to one antimicrobial class was identified in two (4.4%) out of the 45 *C. jejuni* isolates and five (10.4%) of the 48 *C. coli* isolates. Eighteen isolates (19.4%; nine *C. jejuni* and nine *C. coli*) were resistant to two antimicrobial classes. Sixty-three (67.7%) of all the isolates (29 *C. jejuni* and 34 *C. coli*) were classified as MDR, and the rates of resistance profiles that included 3, 4, 5, 6, and 7 antimicrobial classes were 7.5%, 17.2%, 20.4%, 18.3%, and 4.3%, respectively. In terms of the antimicrobial resistance pattern, 23 antimicrobial resistance (AMR) patterns were observed in the *C. jejuni* isolates, whereas 32 AMR patterns were detected in the *C. coli* isolates. The main AMR in *Campylobacter* spp. isolates was the combination of fluoroquinolones, tetracyclines, and macrolides (Table 4).

**TABLE 4:** Multidrug resistance profiles of *C. jejuni* and *C. coli.*

**Antimicrobial agents**	**No. of AMC^∗^**	***C. jejuni* % (*n* = 45)**	***C. coli* % (*n* = 48)**
GEN-CLI-FFN-TET-CIP-NAL-ERY-AZM-TEL	7	6.7(3)	2.1(1)
GEN-CLI-TET-CIP-NA-AZM-ERY-TEL	6	11.1(4)	14.6(8)
GEN-CLI-ERY-TET-AZM-NA-TEL	6	4.4(1)	2.1(2)
CLI-ERY-FFN-TET-CIP-AZM-NA-TEL	6	0(0)	2.1(1)
CLI-ERY-FFN-TET-AZM-NA-TEL	6	0(0)	2.1(1)
CLI-ERY-TET-CIP-AZM-NA-TEL	5	17.8(8)	2.1(1)
GEN-CLI-ERY-TET-CIP-AZM-NA	5	4.4(2)	2.1(1)
GEN-ERY-TET-AZM-NA-TEL	5	2.2(1)	2.1(1)
GEN-ERY-FFN-AZM-NA-TEL	5	0(0)	2.1(1)
CLI-ERY-TET-AZM-NA-TEL	5	0(0)	2.1(1)
CLI-ERY-TET-CIP-NA-TEL	5	0(0)	2.1(1)
CLI-TET-CIP-AZM-NA-TEL	5	2.2(1)	0(0)
GEN-ERY-TET-CIP-AZM-NA-TEL	5	0(0)	2.1(1)
CLI-ERY-TET-CIP-NA	4	4.4(2)	2.1(1)
GEN-ERY-TET-CIP-AZM	4	0(0)	2.1(1)
GEN-CLI-ERY-AZM-NA	4	2.2(1)	0(0)
GEN-CLI-ERY-CIP-AZM	4	0(0)	2.1(1)
CLI-ERY-TET-CIP-AZM-NA	4	0(0)	2.1(1)
CLI-ERY-TET-CIP	4	2.2(1)	0(0)
CLI-TET-CIP-AZM-NA	4	2.2(1)	0(0)
ERY-FFN-TET-CIP-AZM-NA	4	0(0)	2.1(1)
GEN-ERY-AZM-NA-TEL	4	0(0)	2.1(1)
CLI-ERY-CIP-AZM-NA-TEL	4	0(0)	2.1(1)
ERY-TET-CIP-AZM-NA-TEL	4	2.2(1)	2.1(1)
ERY-TET-AZM-NA-TEL	4	0(0)	4.2(2)
GEN-ERY-AZM-NA	3	2.2(1)	0(0)
GEN-TET-CIP-NA	3	0(0)	2.1(1)
GEN-CLI-CIP-NA	3	2.2(1)	0(0)
CLI-ERY-CIP-AZM-NA	3	2.2(1)	2.1(1)
CLI-ERY-CIP	3	0(0)	2.1(1)
ERY-TET-CIP-AZM-NA	3	0(0)	2.1(1)
TET-CIP-NA	2	6.7(3)	8.3(4)
ERY-AZM-NA	2	2.2(1)	4.2(2)
TET-NA	2	4.4(2)	0(0)
CLI-CIP-NA	2	2.2(1)	2.1(1)
CLI-ERY-AZM	2	2.2(1)	0(0)
CLI-ERY	2	0(0)	2.1(1)
ERY-AZM-TEL	2	0(0)	2.1(1)
TET-CIP	2	2.2(1)	0(0)
ERY-AZM	1	0(0)	4.2(2)
ERY	1	0(0)	6.3(3)
TET	1	2.2(1)	0(0)
NA	1	2.2(1)	0(0)
Pan-susceptible	0	11.1(5)	0(0)
MDR (%)		64.4 (29)	64.5 (31)

*AMC, antimicrobial class. CIP, ciprofloxacin; NAL, nalidixic acid; ERY, erythromycin; AZM, azithromycin; TEL, telithromycin; TET, tetracycline; GEN, gentamicin; FFN, florfenicol; CLI, clindamycin.

### Detection of *gyr*A QRDR Mutations Associated With Quinolone Resistance

In this study, all phenotypically ciprofloxacin-and/or nalidixic-acid-resistant strains carried the C257T transition in *gyr*A, which resulted in a T86I amino acid substitution. No mutation at other position was detected in *gyr*A gene.

### Tetracycline Resistance Genes

When we screened for tetracycline resistance genes, the *tet*O gene was detected in all 64 tetracycline-resistant *Campylobacter* spp. strains. The *tet*A gene was present in six (6.45%) isolates, including two *C. coli* and four *C. jejuni*. The *tet*B resistance gene was not detected in any *Campylobacter* isolate.

### Molecular Mechanisms of Macrolide Resistance

The A2075G point mutation within domain V of the 23S rRNA gene was observed in 62 highly macrolide-resistant *Campylobacter* isolates, including 28 *C. jejuni* and 34 *C. coli*. No mutation at other position associated with macrolide resistance was detected in this region in any isolate tested.

Two amino acid changes (V121A and M192I) in ribosomal protein L4 (encoded by the *rpl*D gene) were detected in both macrolide-resistant and macrolide-susceptible *Campylobacter* isolates (Tables 5, 6). Eight macrolide-resistant and four macrolide-susceptible *Campylobacter* isolates carried a V121A substitution in the L4 protein. Nine macrolide-resistant and three macrolide-susceptible *Campylobacter* isolates showed the variant M192I in the L4 protein. Five *Campylobacter* isolates contained both substitutions in the L4 protein.

**TABLE 5:** MIC, presence of *erm*B gene, mutation and insertion of 23S rRNA and L4 and L22 ribosomal protein in *C. jejuni* strains having different resistance to azithromycin and erythromycin^*a*^.

**Strains**	**MIC (μg/ml)**		**23S rRNA^*b*^**	***erm*B**	**Mutations^*c*^**		**Insertions^*c*^**
	**ERY**	**AZM**			**L4**	**L22**	**L22**
CJ-CK-01	> 64	>64	A2075G	**+**	**−**	**−**	**−**
CJ-CK-02	> 64	>64	A2075G	**+**	**−**	**−**	**−**
CJ-CK-03	> 64	>64	A2075G	−	**−**	**−**	**−**
CJ-CK-04	> 64	>64	A2075G	−	**−**	**−**	**−**
CJ-CK-010	> 64	>64	A2075G	**+**	**−**	**−**	**−**
CJ-CK-011	> 64	>64	A2075G	**+**	**−**	**−**	**−**
CJ-CK-018	> 64	>64	A2075G	**−**	**−**	V65I,G74A,A103V,T109S,A111E, A114T,P120T,V121A,X125T,X126S, V130A,A132V,E133K	120TTKAP121
CJ-CK-019	> 64	>64	A2075G	**−**	**−**	V65I,G74A,A103V,T109S,A111E, A114T,P120T,V121A,X125T,X126S, V130A,A132V,E133K	120TTKAP121
CJ-CK-020	> 64	>64	A2075G	**+**	**−**	**−**	**−**
CJ-CK-021	> 64	>64	A2075G	**+**	**−**	**−**	**−**
CJ-CK-057	> 64	>64	A2075G	**+**	**−**	V65I,G74A,T109S,A111E,A114T, V121A,X125T,X126S,V130A,A132V	120TTKAP121
CJ-CK-058	0.12	> 64	A2075G	−	**−**	V65I,G74A,T109S,A111E,A114T,V121A, X125T,X126S,V130A,A132V	120TTKAP121
CJ-CK-061	> 64	>64	A2075G	**+**	**−**	**−**	**−**
CJ-CK-070	> 64	>64	A2075G	−	**−**	**−**	**−**
CJ-CK-071	> 64	>64	A2075G	**+**	**−**	**−**	**−**
CJ-CK-072	32	> 64	A2075G	**+**	**−**	**−**	**−**
CJ-CK-073	> 64	>64	A2075G	**+**	**−**	V65I,G74A,A103V,T109A,A111E,A114T, V121A,X125T,X126S,V130A	120TTKAP121
CJ-CK-074	> 64	>64	A2075G	**+**	**−**	**−**	**−**
CJ-CK-075	> 64	>64	A2075G	**+**	**−**	**−**	**−**
CJ-CK-083	> 64	>64	A2075G	−	V121A	**−**	**−**
CJ-CK-084	> 64	>64	A2075G	−	**−**	**−**	**−**
CJ-CK-087	> 64	>64	A2075G	+	V121A	**−**	**−**
CJ-CK-093	> 64	>64	A2075G	+	**−**	**−**	**−**
CJ-CK-094	> 64	>64	A2075G	+	V121A	**–**	**−**
CJ-CK-097	> 64	>64	A2075G	−	**−**	**−**	**−**
CJ-CK-012	1	0.5	**−**	−	V121A	V65I,G69A,G74A,T109A,A111E,A114T, P120T,V121A,X125T,X126S,V130A,E133K	118APAAKK119,120TTKAP121
CJ-CK-013	2	0.5	**−**	−	V121A	V65I,G69A,G74A,T109A,A111E,A114T, P120T,V121A,X125T,X126S,V130A,E133K	118APAAKK119,120TTKAP121
CJ-CK-046	0.5	2	**−**	−	**−**	V65I,G74A,T109S,A111E,A114T,V121A, X125T,X126S,V130A,A132V	120TTKAP121

*^*a*^CK, chicken; P, pig; Ery, erythromycin; Azi, azithromycin; CJ, C. jejuni. ^*b*^The position of the 23S rRNA gene mutation. ^*c*^The positions of the *rplD* and *rplV* genes encoding L4 and L22 ribosomal proteins changes.*

**TABLE 6:** MIC, presence of *erm*B gene, mutation and insertion of 23S rRNA and L4 and L22 ribosomal protein in *C. coli* strains having different resistance to azithromycin and erythromycin^*a*^.

**Strains**	**MIC (μg/ml)**		**23S rRNA^*b*^**	***erm*B**	**Mutations^*c*^**		**Insertions^*c*^**
	**ERY**	**AZM**			**L4**	**L22**	**L22**
CC-P-022	> 64	>64	A2075G	+	**−**	**−**	**−**
CC-P-023	> 64	>64	A2075G	+	M192I	**−**	**−**
CC-P-024	> 64	>64	A2075G	−	**−**	**−**	**−**
CC-P-025	> 64	>64	A2075G	−	**−**	**−**	**−**
CC-P-026	> 64	>64	A2075G	−	**−**	**−**	**−**
CC-P-027	> 64	>64	A2075G	−	**−**	**−**	**−**
CC-P-028	> 64	>64	A2075G	−	**−**	**−**	**−**
CC-P-029	> 64	>64	A2075G	−	V121A, M192I	**−**	**−**
CC-P-030	> 64	>64	A2075G	−	V121A,	A103V	**−**
CC-P-031	> 64	>64	A2075G	−	M192I	**−**	**−**
CC-P-032	> 64	>64	A2075G	−	M192I	**−**	**−**
CC-P-033	> 64	>64	A2075G	−	**−**	**−**	**−**
CC-P-034	> 64	>64	A2075G	−	**−**	**−**	**−**
CC-P-035	> 64	1	A2075G	−	**−**	**−**	**−**
CC-P-036	> 64	>64	A2075G	−	M192I	A103V,T109A,A111E,A114T,P120T, V121A,X125T,X126S	120TTKAP121
CC-P-037	> 64	1	A2075G	−	V121A, M192I	A103V	**−**
CC-P-038	> 64	>64	A2075G	−	**−**	**−**	**−**
CC-P-039	> 64	4	A2075G	−	M192I	**−**	**−**
CC-P-040	> 64	>64	A2075G	−	M192I	**−**	**−**
CC-P-042	> 64	>64	A2075G	−	V121A, M192I	A103V	**−**
CC-P-043	> 64	1	A2075G	−	V121A, M192I	A103V	**−**
CC-P-044	> 64	>64	A2075G	+	**−**	**−**	**−**
CC-P-045	> 64	>64	A2075G	−	M192I	**−**	**−**
CC-CK-049	> 64	>64	A2075G	+	**−**	**−**	**−**
CC-CK-050	> 64	>64	A2075G	−	**−**	**−**	**−**
CC-CK-051	> 64	32	A2075G	+	**−**	**−**	**−**
CC-CK-066	> 64	>64	A2075G	+	**−**	**−**	**−**
CC-CK-069	> 64	>64	A2075G	−	**−**	**−**	**−**
CC-CK-076	> 64	>64	A2075G	+	**−**	**−**	**−**
CC-CK-077	> 64	>64	A2075G	+	**−**	**−**	**−**
CC-CK-078	> 64	>64	A2075G	+	**−**	**−**	**−**
CC-CK-079	> 64	>64	A2075G	+	**−**	**−**	**−**
CC-CK-081	> 64	>64	A2075G	+	**−**	**−**	**−**
CC-CK-095	> 64	>64	A2075G	−	**−**	**−**	**−**
CC-CK-096	> 64	>64	A2075G	−	**−**	**−**	**−**
CC-CK-099	64	8	A2075G	−	**−**	**−**	**−**
CC-CK-100	64	16	A2075G	−	**−**	**−**	**−**
CC-CK-054	0.5	1	−	+	V121A	**−**	**−**
CC-CK-067	1	0.06	−	−	V121A, M192I	**−**	**−**

*^*a*^CK, chicken; P, pig; ERY, erythromycin; AZM, azithromycin; CC, *C. coli*. ^*b*^The position of the 23S rRNA gene mutation. ^*c*^The positions of the *rpl*D and *rpl*V genes encoding L4 and L22 ribosomal proteins changes.*

Fifteen amino acid substitutions in the L22 ribosomal protein were observed: V65I (*n* = 8), G69A (*n* = 2), G74A (*n* = 8), A103V (*n* = 8), T109A (*n* = 4), T109S (*n* = 5), A111E (*n* = 9), A114T (*n* = 9), P120T (*n* = 5), V121A (*n* = 9), X125T (*n* = 9), X126S (*n* = 9), V130A (*n* = 8), E133K (*n* = 4), and A132V (*n* = 5). The L22 protein of the tested isolates also contained two amino acid insertions (118APAAKK119 and 120TTKAP121) relative to the L22 protein of *C. jejuni* NCTC 11168 (Tables 5, 6).

The *erm*B gene was identified in 49 isolates (52.7%, 49/93): 30 *C. jejuni* (66.7%) and 19 *C. coli* (39.6%). The *erm*B-positive rate in the chicken-origin *Campylobacter* spp. isolates (65.7%, 46/70) was significantly higher than that in the *Campylobacter* isolates with a pig origin (13.0%, 3/23). Notably, only one *C. jejuni* isolate showing high level macrolide resistance did not contain the 23S rRNA mutation, but did carry the *erm*B gene. Twenty-seven *erm*B-positive isolates also contained the A2075G 23S rRNA mutation (Tables 5, 6).

## Discussion

Thermophilic *Campylobacter* are zoonotic pathogens, and antimicrobial resistance in *Campylobacter* has gradually become a major public health problem in both developed and developing countries (Wieczorek and Osek, 2013). In the present study, 45 *C. jejuni* and 25 *C. coli* were isolated from chickens, and 23 *C. coli* were isolated from pigs. The *C. jejuni* was the predominant *Campylobacter* species in chicken isolates (64.3%), followed by *C. coli* (35.7%), and *C. coli* was the dominant *Campylobacter* species in pigs, accounting for 100% of the pig isolates. The isolation rates of *Campylobacter* on pig farms was 23.0%, which was within the range of 13.4%–26.1% reported by Wang et al. (2016). In China, laying hens are used as stewed chicken. Hence, *Campylobacter* in layers can also pose a risk for consumers. The isolation rates of *Campylobacter* on layer farms (34.3%) was lower than the rate on broiler farms (57.5%). The total isolation rates of *Campylobacter* on chicken farms in Jiangsu Province was 46.7%, which was higher than the isolation rates in other regions of China (Wang et al., 2016). The isolation rate of *Campylobacter* differed in different regions, which may be attributable to the different prevalence of *Campylobacter* in different regions or to different animal breeding conditions, breeding scales, sample numbers, and the isolation methods used by various laboratories.

Telithromycin belongs to a class of semisynthetic macrolides, the ketolides, and has been designed to treat respiratory infections. In this study, resistance to telithromycin was highly prevalent among the *Campylobacter* isolates: 42.2% of *C. jejuni* isolates from chickens, 44.0% of *C. coli* isolates from chickens, and 60.9% of *C. coli* isolates from pigs were resistant to telithromycin. We noted a difference in telithromycin resistance between *C. coli* from chickens and those from pigs, but the relationship between the emergence of ketolide resistance among *Campylobacter* and the macrolides used in pig production is not yet clear. The resistance rate to gentamicin in the *C. coli* isolates from chickens (40.0%) was similar to that for *C. jejuni* isolates (31.1%). These findings are inconsistent with the results of Li et al. (2016), who reported that the prevalence of gentamicin resistance among *C. coli* isolates was 93.6%, whereas that in *C. jejuni* isolates was 17.9%. Overall, our results indicate that the drug resistance rates in *C. coli* for the antimicrobial classes tested were higher than those in *C. jejuni* from chickens. In recent years, MDR *Campylobacter* strains have been a growing global public health problem. In this study, a high prevalence of MDR *Campylobacter* isolates was detected (up to 64.5%). However, the MDR rate was lower than in other countries, such as Italy (100%) (Fraqueza et al., 2014). The prevalence of MDR isolates of *C. coli* (68%) was similar to that of *C. jejuni* (64.4%) in chickens, which is inconsistent with the results of Wang et al. (2016). The reason for this discrepancy may be the small number of samples examined in the present study.

High rates of fluoroquinolone resistance were observed among the 93 *Campylobacter* isolates. We found that the rates of fluoroquinolone resistance in *C. coli* (92.0% and 100%) and *C. jejuni* isolates (80.0% and 66.7%) from chickens were higher than the rates of resistance in *C. coli* isolates from pigs (65.2% and 21.7%). This was attributable to the use of fluoroquinolones as the preferred drugs against bacterial infections in poultry production, and has been caused by the unreasonable and uncontrolled use of antimicrobial agents in poultry production in China. These results are consistent with previous reports in China, in which the *Campylobacter* strains isolated from chickens were resistant to quinolones (Ma et al., 2014; Li et al., 2016). Therefore, the high prevalence of fluoroquinolone-resistant *Campylobacter* isolates in chickens may cause serious public health problems.

Quinolone resistance in *Campylobacter* spp. is usually caused by the point mutation T86I in the gyrase protein, and is the most frequently detected mechanism (Hormeño et al., 2016). In this study, 79 *Campylobacter* isolates (39 *C. jejuni* and 40 *C. coli*) with the quinolone (ciprofloxacin or nalidixic acid) resistance phenotype were positive for the T86I mutation. Other authors have reported similar results, where all ciprofloxacin-resistant *Campylobacter* strains carried the same mutation (Woźniak-Biel et al., 2018; Elhadidy et al., 2020).

Given the long-term use of tetracyclines in feed additives for livestock and poultry, large numbers of tetracycline-resistant isolates are found in animal reservoirs (Premarathne et al., 2017; Hafez and Attia, 2020). It has previously been reported that 95.6% of *C. jejuni* isolates from chickens, 97.5% of *C. coli* isolates from chickens and 97.5% of *C. coli* isolates from pigs in China were resistant to tetracycline (Wang et al., 2016). In the present study, a relatively high level of resistance to tetracycline was observed in the *C. jejuni* (71.1%) and *C. coli* (88.0%) isolates from chickens.

The *tet*O gene is the most commonly reported tetracycline resistance gene in *Campylobacter* spp. (Luangtongkum et al., 2009), and is located on the chromosome or transmissible plasmids (Abdi-Hachesoo et al., 2014; Narvaez-Bravo et al., 2017). In this study, all 64 tetracycline-resistant *Campylobacter* isolates (32 *C. jejuni* and 32 *C. coli*) carried the *tet*O gene. The high prevalence of the *tet*O gene reflects the high rate of tetracycline resistance in *Campylobacter* spp. isolated from chickens and pigs in China.

Chopra and Roberts (2001) reported that the efflux proteins encoded by *tet*A and *tet*B can export tetracycline from the cell. In this study, the *tet*A detection rate in the *Campylobacter* isolates was 6.5%, but there was no evidence of *tet*B. Abdi-Hachesoo et al. (2014) reported detecting the *tet*A resistance gene in 18% of *Campylobacter* spp. isolated from poultry carcasses in Iran. Nguyen et al. (2016) reported that the detection rates of *tet*A gene in *C. jejuni* and *C. coli* isolates from chickens in Kenya were 90.3% and 100%, respectively. Previous studies have proved that the *tet*A gene can be located on mobile elements, such as plasmids, and can be horizontally transferred among bacterial strains (Szczepanowski et al., 2004). *Campylobacter* spp. with the *tet*A gene may be involved in the distribution of this resistance gene to other food-borne bacteria in the animal industry.

The prevalence of *Campylobacter* resistance to macrolides varies according to geographic region (Rożynek et al., 2013). In the present study, erythromycin and azithromycin resistance were detected in 62.2%–100.0% and 60.0%–82.6% of *Campylobacter* isolates from pigs and chickens. In Korea in 2010, Lim *et al.* reported that 15.5% of *Campylobacter* isolates from chicken and swine feces or carcasses showed phenotypic resistance to erythromycin (Lim et al., 2017). Rozynek et al. reported a low rate of erythromycin resistance (4.7%), which was similar to that reported in chicken-origin isolates in other European countries (Rożynek et al., 2013). In the present study, the rates of erythromycin and azithromycin resistance were much higher in *C. coli* from chickens and pigs than in *C. jejuni* from chickens, as has been observed in previous studies (Chen et al., 2010; Wang et al., 2016). The rate of erythromycin-resistant *C. coli* isolates in pigs (100%) was higher than the rate in chickens (72.0%), which was much higher than has been reported in previous studies in China (Qin et al., 2011). Notably, in the present study, the erythromycin resistance rate in *C. jejuni* from chickens (62.2%) was higher than those reported in most previous studies undertaken in China (Chen et al., 2010; Li et al., 2016; Wang et al., 2016; Zhang et al., 2016). High levels of macrolide resistance were attributable to the widespread and increasing use of macrolides in animal production, including tylosin, tilmicosin, erythromycin, and kitasamycin.

When the macrolide-resistant isolates were screened, a range of different molecular resistance mechanisms were identified, including mutations in the 23S rRNA, *rpl*D, and *rpl*V genes and the presence of *erm*B. Our results show that the A2075G mutation in the 23S rRNA gene in the *Campylobacter* isolates might be the cause of their high resistance to azithromycin and erythromycin. The rate of the A2075G mutation in the present study was 98.4% (62/63) in the *Campylobacter* isolates with high level resistance to erythromycin, which is similar to the frequencies reported in a previous study (Luangtongkum et al., 2009). The mutation was not present in *Campylobacter* isolates that were susceptible to azithromycin or erythromycin or that displayed low level or intermediate resistance to these agents. No other mutations in the 23S rRNA gene were detected in this study.

Several studies have reported that ribosomal protein L4 mutations at amino acid positions 192 and 121, which conferred no significant difference between macrolide-resistant and -susceptible strains (Lehtopolku et al., 2011; Qin et al., 2011). In the present study, several mutations, including V65I, G74A, A103V, T109A, T109S, A111E, A114T, V130A, and A132V, in ribosomal protein L22 were detected in macrolide-susceptible and macrolide-resistant strains. This result is similar to a previous report by Lim et al. (2017). However, in the present study, several mutations are reported for the first time, including G69A, P120T, V121A, X125T, X126S, and E133K, which were found in both macrolide-susceptible and -resistant isolates. We noted one amino acid insertion (118APAAKK119) in the L22 ribosomal protein in nine *Campylobacter* isolates and another amino acid insertion (120TTKAP121) in L22 in two macrolide-susceptible *C. jejuni* isolates. The L22 protein of *C. jejuni* 81-176 also contains amino acid insertion 118APAAKK119 (Hao et al., 2013). Our results suggested that these changes were unlikely to contribute directly to macrolide resistance.

The mechanism of resistance mediated by *erm*B is particularly noteworthy because this gene can transfer macrolide resistance horizontally among *Campylobacter* strains and confer high-level resistance (Qin et al., 2014). In the present study, the detection rate of *erm*B was 52.7%, which was higher than those reported in other areas of China (Wang et al., 2014; Li et al., 2016). We observed that the rate of *erm*B-positive *C. jejuni* isolates (30/45) was significantly higher than that of *erm*B-positive *C. coli* isolates (19/48, *P* = 0.0089), which is inconsistent with previous reports (Zhang et al., 2016; Liu et al., 2017). The prevalence of the *erm*B gene in the *Campylobacter* from chickens (65.7%, *n* = 46) was significantly higher than in those from pigs (13.0%, *n* = 3, *P* < 0.0001). Notably, 27 (43.5%, 27/62) *erm*B-positive isolates also carried the A2075G mutation in 23S rRNA, which is a higher rate than in previous reports by Wang et al. (38%, 22/58) (Wang et al., 2014) and Zhang *et al.* (1.9%, 3/157) (Zhang et al., 2016). These results confirmed that *ermB* was markedly more prevalent among *Campylobacter* strains isolated from chickens than in those from pigs. The *erm*B gene must be monitored in *Campylobacter* because it is so highly transmittable. In general, the A2075G point mutation in domain V of the 23S rRNA and the *erm*B gene were the main causes of macrolide resistance in the *Campylobacter* isolates from Jiangsu Province, China.

## Conclusion

In conclusion, this study has demonstrated that *Campylobacter* spp. isolated from chickens and pigs had high drug resistance rates to fluoroquinolones, tetracyclines and macrolides. The emergence of MDR *Campylobacter* strains is attributable to the widespread use of antibiotics in poultry and pig production. Monitoring antimicrobial resistance in *Campylobacter* and the prudent use of antimicrobials in animal-food production are essential to reducing antimicrobial resistance in bacterial pathogens.

## Data Availability Statement

All datasets generated for this study are included in the article/supplementary material.

## Ethics Statement

The animal study was reviewed and approved by Animal Welfare and Ethical Censor Committee at Jiangsu Institute of Poultry Science. Written informed consent was obtained from the owners for the participation of their animals in this study.

## Author Contributions

QZ, JZ, XZ, and MT performed antibiotic susceptibility tests. XT and JL collected the samples. XT, MT, and SZ did PCR amplification and DNA sequence analysis. QZ conducted the statistical analysis of data. MT completed the manuscript. YG directed the study and assisted in the completion of the manuscript. All authors read and agreed to submit the manuscript.

## Conflict of Interest

The authors declare that the research was conducted in the absence of any commercial or financial relationships that could be construed as a potential conflict of interest.
